# Ageing and smoking contribute to plasma surfactant proteins and protease imbalance with correlations to airway obstruction

**DOI:** 10.1186/1471-2466-11-19

**Published:** 2011-04-19

**Authors:** Helen Ilumets, Witold Mazur, Tuula Toljamo, Noora Louhelainen, Pentti Nieminen, Hideo Kobayashi, Nobuhisa Ishikawa, Vuokko L Kinnula

**Affiliations:** 1 Department of Medicine, Division of Pulmonary Medicine, University of Helsinki and Helsinki University Central Hospital, Helsinki, Finland; 2 Department of Medicine, Division of Pulmonary Medicine, Lapland Central Hospital, Rovaniemi, Finland; 3 Medical informatics and statistics research group, University of Oulu, Oulu, Finland; 4 Department of Medicine, Division of Pulmonary Medicine, National Defense Medical College, Tokorozawa, Japan; 5 Departments of Molecular and Internal Medicine, Graduate School of Biomedical Science, Hiroshima University, Hiroshima, Japan

## Abstract

**Background:**

A significant number of young people start smoking at an age of 13-15, which means that serious smoking-evoked changes may have been occurred by their twenties. Surfactant proteins (SP) and matrix metalloproteinases (MMPs) and their tissue inhibitors (TIMPs) have been linked to cigarette smoke induced lung remodelling and chronic obstructive pulmonary disease (COPD). However, the level of these proteins has not been examined during ageing or in young individuals with short smoking histories.

**Methods:**

Plasma levels of SP-A, SP-D, MMP-9, and TIMP-1 were measured by EIA/ELISA from young (18-23 years) non-smoking controls (YNS) (n = 36), smokers (YS) (n = 51), middle aged/elderly (37-77 years) non-smoking controls (ONS) (n = 40), smokers (OS) (n = 64) (FEV1/FVC >0.7 in all subjects) and patients with COPD (n = 44, 35-79 years).

**Results:**

Plasma levels of SP-A increased with age and in the older group in relation to smoking and COPD. Plasma SP-D and MMP-9 levels did not change with age but were elevated in OS and COPD as compared to ONS. The TIMP-1 level declined with age but increased in chronic smokers when compared to ONS. The clearest correlations could be detected between plasma SP-A vs. age, pack years and FEV1/FVC. The receiver operating characteristic (ROC) curve analysis revealed SP-A to be the best marker for discriminating between patients with COPD and the controls (area under ROC curve of 0.842; 95% confidence interval, 0.785-0.899; p < 0.001).

**Conclusions:**

Age has a significant contribution to potential markers related to smoking and COPD; SP-A seems to be the best factor in differentiating COPD from the controls.

## Background

Smoking is the major risk factor for the development of chronic obstructive pulmonary disease (COPD), and smoking cessation is the only effective way to slow down disease progression [1-3]. Young people generally start smoking at 13-15 years of age, which means that significant changes due to smoking may have been occurred within 10 years i.e. by the time they are 25 years, they may have suffered the damage which will later develop into COPD. There is a need to devise sensitive and specific markers for early COPD, but at present, the tests are unreliable. In this study, we measured plasma levels of surfactant protein A (SP-A), surfactant protein D (SP-D), matrix metalloproteinase-9 (MMP-9) and tissue inhibitor of matrix metalloproteinase-1 (TIMP-1) in young and middle aged/elderly smokers and in patients with COPD. The selection of these potential marker compounds was based on previous studies on COPD, i.e. either non-hypothesis/proteomics (SP-A) or hypothesis driven (SP-D, MMP-9) studies, which have indicated that especially these markers may predict COPD, its development and/or progression [4-9]. These compounds have never been compared previously in one single investigation.

Pulmonary surfactant is a mixture of phospholipids and proteins formed mainly by type II pneumocytes [10]. SP-A and SP-D are members of the collectin family and play important and unique roles in pulmonary defense against inflammation/oxidative stress [11,12]. The surfactant composition and functions have been found to be modulated by smoking and/or COPD [13-16], and most studies in this field have reported elevated levels of SP-A in the serum of the patients with COPD [6,8]. Serum SP-D has been postulated to be a potential marker for COPD, being able to predict both exacerbations and response to corticosteroid therapy [17-20]. Surfactants have also been found to regulate the balance of proteases/antiproteases through different pathways, SP-A may even regulate MMP-9 production and function [21-24]. One of the most widely suggested hypothesis in the pathogenesis of COPD involves an imbalance between proteases and antiproteases [4,25].

Matrix metalloproteinases cleave components of the extracellular matrix and basement membranes and the balance of their activities is strictly controlled by their inhibitors. The changes in these proteases, especially MMP-9 and its major endogenous inhibitor TIMP-1, have been strongly linked to smoking and COPD [4,5,26,27]. However, little is known about whether there are age related alterations in these proteins in smokers and/or COPD.

The main goal of this study was to find out whether smoking and ageing affect the levels and relationships between circulating SP-A, SP-D, MMP-9 and TIMP-1.

The second purpose was to determine whether the levels of these markers would be associated with demographic parameters and lung function values in young and middle aged/elderly smokers, as well as in COPD patients in comparison to their age-matched controls. None of the subjects had any other environmental exposures, all COPD cases were newly diagnosed, and had no other diagnosed diseases nor were they taking any medications.

## Methods

### Subjects and samples

Plasma samples were collected from middle aged/elderly subjects who had been contacted from the Division of Pulmonary Medicine, Lapland Central Hospital [28] and from young smokers and non-smokers who were military draftees from the Northern Command of the Finnish Defence Forces [29]. Details of these cohorts have been described in the abovementioned studies [28,29]. Based on self-reported detailed questionnaire all subjects were symptom-free and considered themselves as healthy, they had no other environmental exposures (such as second hand smoke, pollutants or asbestos fibres) [28]. The study population included young (age < 25 years) healthy smokers (YS), young non-smoking healthy controls (YNS), middle-aged/elderly healthy smokers (OS) and non-smoking healthy controls (ONS), and patients with stable COPD (Stage I-III). The diagnosis of COPD was defined according to the Global Strategy for the diagnosis, management and prevention of COPD (GOLD) criteria; FEV_1 _< 80% of predicted, FEV_1_/FVC < 70% and bronchodilatation effect < 12% [2,30]. Spirometric values were assessed by standard spirometry (Medriko M 904, Kuopio, Finland) and the Finnish reference values for spirometry [31]. Exclusion criteria included allergies, asthma, a history of respiratory disease, or respiratory infection less than 8 weeks before entering the study. None of the subjects had any other diagnosed disease or any medications. The study was approved by the Ethics Committee of Lapland Central Hospital (4^th ^June 2003 and 31^st ^October 2006) and all participants provided written informed consent.

### The measurement of SP-A, SP-D, MMP-9 and TIMP-1

Plasma samples were randomly chosen from the cohorts that represented the various sub-groups for the study. SP-A and SP-D levels were measured by commercially available EIA/ELISA kits (SP-A test Kokusai-F kit, Sysmex, Kobe, Japan; SP-D kit Yamasa EIA kit, Yamasa Co., Chiba, Japan) as described [6,32]. MMP-9 and TIMP-1 levels were determined by commercially available ELISA kits (Amersham Biosciences, Cardiff, UK) according to the manufacturers' instructions. The detection limits of SP-A and SP-D were 1.0 ng/ml and 17.2 ng/ml, respectively and for MMP-9 and TIMP-1 0.6 ng/ml and 1.25 ng/ml, respectively.

### Statistical analysis

The data are given as means together with standard deviation (SD). All statistical analyses were performed with the SPSS 18.0 software program (SPSS Inc., Chicago, IL). The analyses of variance (ANOVA) and t-test for independent groups were used to evaluate the statistical significances between the study groups. Linear multivariate regression analysis was used to study the independent effect of age, smoking status and COPD to SP-A, SP-D, MMP-9 and TIMP-1. Correlations between the variables were determined with the Pearson correlation coefficient. Due to the pair wise comparisons, a p-value of <0.01 was considered statistically significant. SP-A, SP-D, MMP-9 and TIMP-1 levels were further analyzed for their predictive capability to distinguish patients with COPD from the control subjects (YNS and ONS) according to receiver operating characteristic (ROC) curves.

## Results

### Subjects and lung function characteristics

The characteristics of the subjects are shown in Table 1. All young and middle aged/elderly smokers and non-smokers had normal airway function according to the GOLD criteria (post bronchodilator FEV_1_/FVC > 0.7) [30]. In the COPD group, there were 15 patients with stage I, 24 patients with stage II and 5 patients with stage III COPD according to the GOLD classification. All COPD cases were newly diagnosed and were taking no regular medications.

**Table 1:** The characteristics of the patients

Variable	Young		Middle aged/elderly		
	
	Non-smokers	Smokers	Non-smokers	Smokers	COPD
**Number**	36	51	40	64	44
**Age (yr)**	19.5 (0.8)	20.0 (1.0)	53.3 (8.8)	52.1 (8.0)	61.3 (8.5)
**Sex (F/M)**	2/34	1/50	28/12	24/40	9/35
**Pack-years**	-	5.1 (2.3)	-	30.4 (14.3)	39.8 (15.0)
**BMI**	24.1 (2.5)	24.1 (3.1)	26.5 (4.2)	27.7 (4.0)	26.9 (3.8)
**Post- bronchodilator** FVC (l)	5.5 (0.8)	5.5 (0.8)	3.8 (0.8)	4.0 (0.9)	3.8 (0.9)
FEV1 (l)	4.8 (0.7)	4.8 (0.7)	3.2 (0.6)	3.3 (0.7)	2.3 (0.7)
FEV1 (% predicted)	97.3 (6.1)	100.1 (9.5)	104.7 (13.6)	95.3 (12.2)	70.0 (16.8)
FEV1/FVC	87.6 (5.6)	87.1 (4.8)	84.5 (5.9)	82.7 (5.1)	60.6 (9.3)

COPD: chronic obstructive pulmonary disease; BMI: body mass index; FVC: forced vital capacity; FEV_1_: forced expiratory volume in one second. Data is shown as mean (SD).

### The level of SP-A increases with ageing, smoking, and COPD

**Figure 1:**
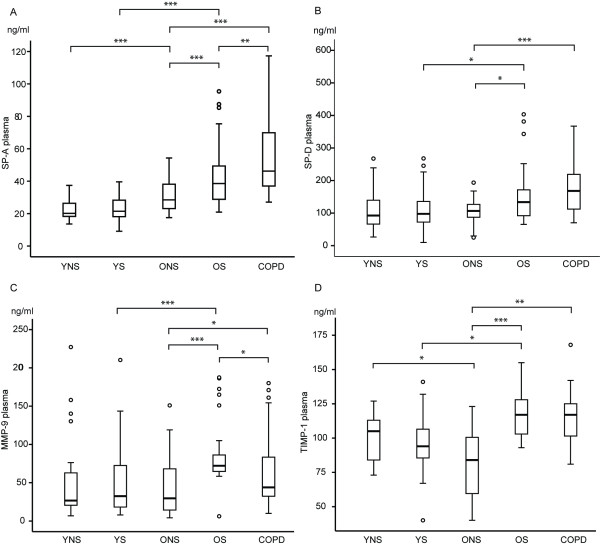
**Box-and-whisker plots of SP-A, SP-D, MMP-9 and TIMP-1 in plasma (A, B, C and D, respectively)**. The boxes represent the 25th to 75th percentiles, the solid lines within the boxes show the median values, the whiskers are the 10th and 90th percentiles, and the points represent outliers. *: p < 0.05; **: p < 0.01; ***: p < 0.001 (between two groups, t-test).

Plasma level of SP-A increased with age in non-smokers and smokers (p < 0.0001 and p < 0.0001), respectively. In young non-smokers (YNS), the mean values were 22.2 ± 6.0 ng/ml, and in middle aged/elderly non-smokers they were 31.4 ± 10.6 ng/ml, the corresponding mean values in YS being 23.0 ± 6.8 ng/ml and OS 43.3 ± 17.5 ng/ml. This latter finding may be partly related to the longer smoking history of OS. Cigarette smoke had no significant effect on the SP-A levels in the young age group, with their relatively short smoking histories. In the older age group, plasma SP-A was higher in OS when compared to ONS (p < 0.0001). Importantly, plasma SP-A was also higher in COPD (55.4 ± 24.6 ng/ml) compared to OS (p = 0.009), and ONS (p < 0.0001) (Figure 1A). The linear regression analysis confirmed that age (regression coefficient (B) = 6.01, standard error (SE) = 2.54, p = 0.019), cigarette smoking (B = 0.45, SE = 0.07, p < 0.001) and COPD (B = 17.08, SE = 3.67, p < 0.001) had independent effects on SP-A level.

### The level of SP-D does not change with ageing but is elevated in OS and COPD compared to ONS

Plasma level of SP-D did not change with age, i.e. the mean levels in YNS and ONS (109.0 ± 62.3 ng/ml and 106.0 ± 41.2 ng/ml) were similar. Furthermore there was no difference between YNS and YS. In the older group, the levels were higher in OS (156.2 ± 90.2 ng/ml) and COPD (186.3 ± 101.9 ng/ml) when compared to ONS (p = 0.012 and p < 0.0001) but there was no significant difference between OS and COPD (Figure 1B). COPD patients had even higher levels of SP-D (B = 64.72, SE = 19.53, p = 0.001) when adjusted for age with linear multivariate regression analysis.

### The level of MMP-9 is elevated in OS and COPD compared to ONS

The plasma concentrations of MMP-9 were very similar in YNS and ONS (47.1 ± 49.1 and 44.4 ± 34.6 ng/ml) and there was no significant difference between YNS and YS. In the older age group, the levels were higher in OS (86.5 ± 40.8 ng/ml) compared to ONS (p < 0.0001). The level of MMP-9 was significantly higher in COPD than in ONS (p = 0.033). The MMP-9 results are summarized in Figure 1C. Linear regression analysis revealed that both age (B = 0.44, SE = 0.19, p = 0.022) and cigarette smoking (B = 26.0, SE = 6.91, p < 0.0001) had independent effects on the MMP-9 level.

### The levels of TIMP-1 and MMP-9/TIMP-1 increase in response to long-term smoking

The plasma level of TIMP-1 declined with age (p = 0.03), but there was no significant difference between YNS and YS. In the older group, the TIMP-1 levels were higher in OS (117.7 ± 18.2 ng/ml) and COPD (114.9 ± 23.5 ng/ml) than in ONS (81.3 ± 26.6 ng/ml, p < 0.001 and p = 0.001), but there was no significant difference between OS and COPD (Figure 1D). The ratio of MMP-9 to TIMP-1 did not change with age. In addition, there were no significant differences between YNS and YS. In the older group, the MMP-9/TIMP-1 ratio was higher in OS (0.79 ± 0.3) than in ONS (0.28 ± 0.2, p < 0.0001) indicative of a significant protease/antiprotease imbalance in favor of proteases. In addition, the linear regression analysis confirmed the independent effect of smoking on TIMP-1 (B = 16.18, SE = 6.66, p = 0.018) and MMP-9/TIMP-1 (B = 4.01, SE = 0.15, p = 0.014).

### Significant correlations can be seen especially with SP-A

**Figure 2:**
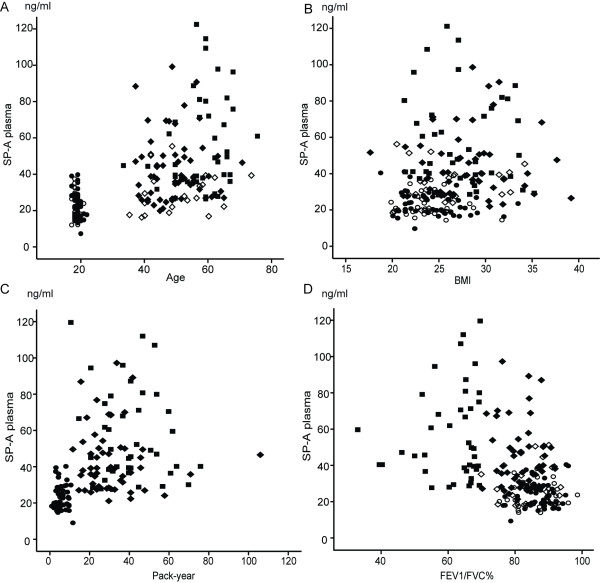
**Relationship between plasma SP-A levels and the demographic parameters, age (A), BMI (B), pack-years (C) and FEV1/FVC% of predicted (D), in all of the subjects**. ○ = young non-smokers; ● = young smokers; ◊ = middle aged/elderly non-smokers; ♦ = middle aged/elderly smokers; ■ = COPD patients.

**Figure 3:**
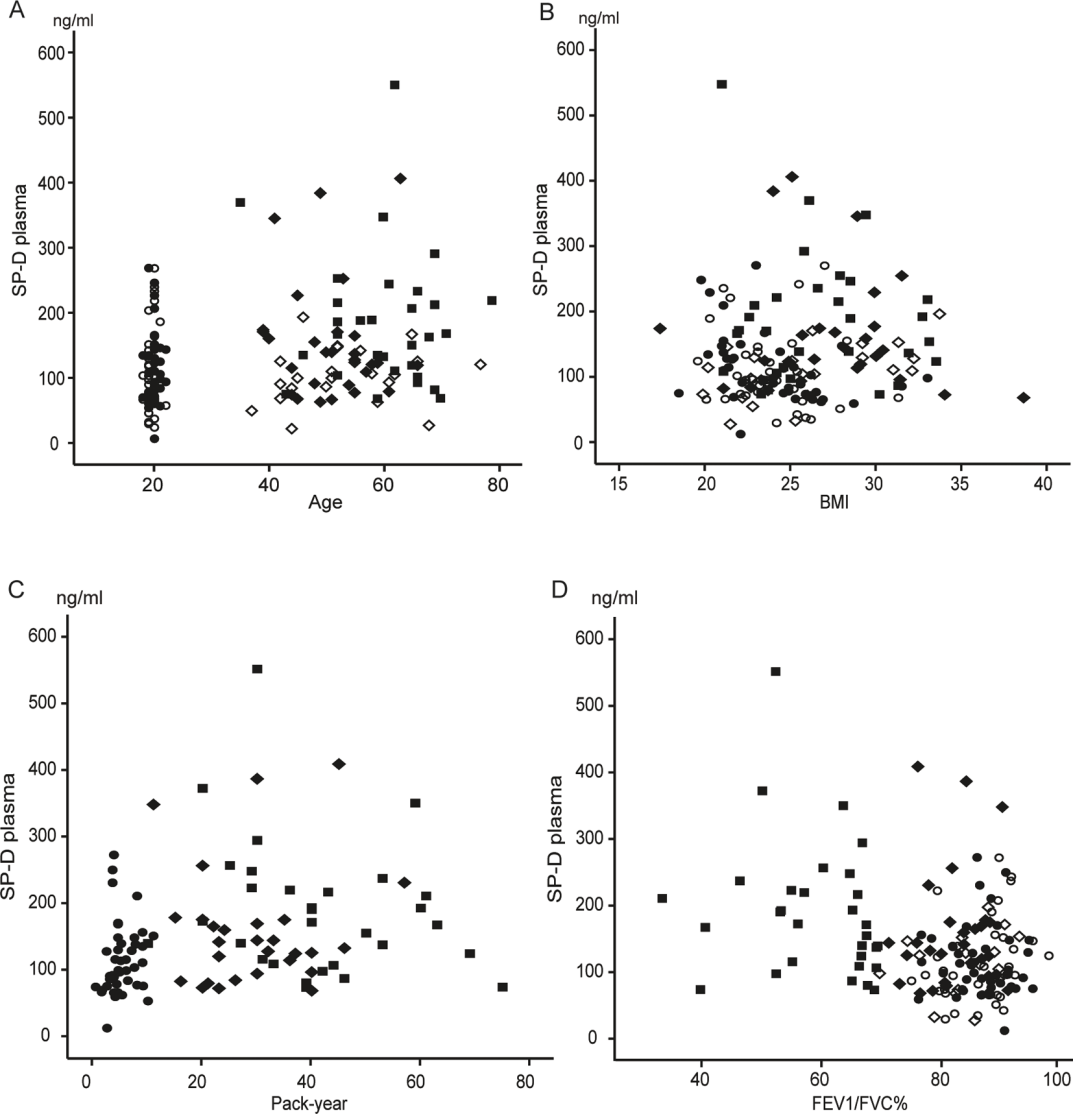
**Relationship between plasma SP-D levels and the demographic parameters, age (A), BMI (B), pack-years (C) and FEV1/FVC% of predicted (D), in all of the subjects**. ○ = young non-smokers; ● = young smokers; ◊ = middle aged/elderly non-smokers; ♦ = middle aged/elderly smokers; ■ = COPD patients.

**Figure 4:**
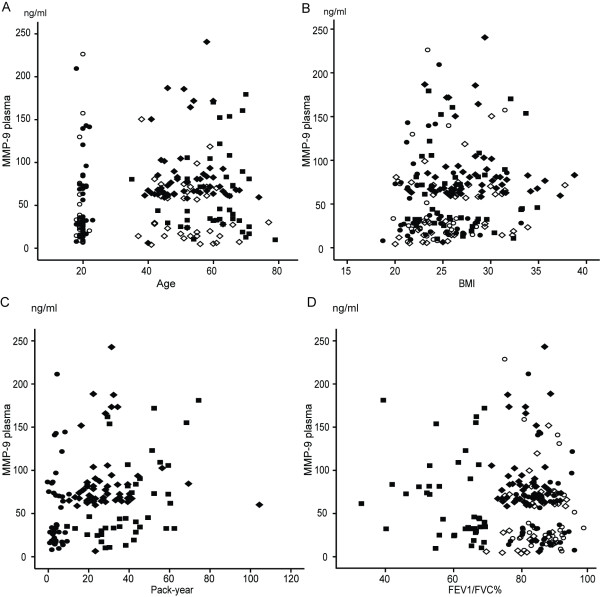
**Relationship between plasma MMP-9 levels and the demographic parameters, age (A), BMI (B), pack-years (C) and FEV1/FVC% of predicted (D), in all of the subjects**. ○ = young non-smokers; ● = young smokers; ◊ = middle aged/elderly non-smokers; ♦ = middle aged/elderly smokers; ■ = COPD patients.

Figures 2, 3 and 4 show how SP-A, SP-D and MMP-9 are related to age, pack years, BMI and lung functions in different study groups. The clearest correlations could be seen with plasma SP-A vs. age, pack years and FEV1/FVC (Figure 2A-D), though some correlations could also be observed with SP-D (Figure 3A-D). The plasma MMP-9 level correlated only with age and pack years, though some minor correlations could be seen with all these parameters and BMI (Figure 4A-D). The levels of SP-A also correlated with SP-D (r = 0.36, p < 0.0001).

### Receiver Operating Characteristic (ROC) curve analysis is most accurate for SP-A

**Figure 5:**
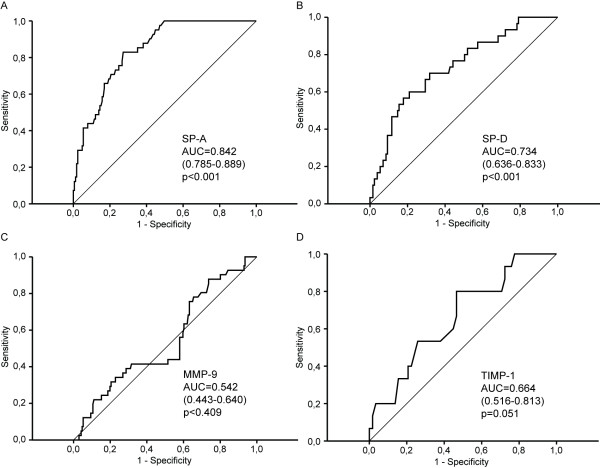
**Receiver operating characteristic curves of SP-A, SP-D, MMP-9 and TIMP-1 in plasma (A, B, C and D, respectively) obtained from patients with COPD, young and middle aged/elderly smokers and the controls**. Values in parentheses indicate 95% confidence intervals for the area under the curve (AUC).

ROC curve analysis was carried out to evaluate the sensitivity, specificity and diagnostic accuracy of plasma SP-A, SP-D, MMP-9 and TIMP-1. The areas under the ROC curve were as follows: for SP-A: 0.845 (95% confidence interval (CI), 0.787 to 0.902, p < 0.001); for SP-D: 0.734 (95% CI, 0.636 to 0.883, p < 0.001); for MMP-9: 0.551 (95% CI, 0.450 to 0.652, p = 0.320) and for TIMP-1: 0.664 (95% CI, 0.516 to 0.813, p = 0.051) (Figure 5).

## Discussion

The present study evaluated plasma levels of SP-A, SP-D, MMP-9 and TIMP-1 in young and middle aged/elderly non-smokers and smokers, and patients with COPD. All of these putative markers have been suggested to associate with COPD. When these potential parameters were evaluated, SP-A appeared to be the most promising marker for COPD since its levels were elevated after long-term smoking and also in COPD as compared to the situation in chronic smokers. SP-A was also clearly correlated with age, pack years and airway obstruction. However, it remains unclear whether SP-A can be used as a marker for COPD or its development. The levels of none of these proteins were changed due to smoking in the group of young smokers who had relatively short smoking histories, no diseases or other exposures and normal values in spirometry.

The elevation of plasma SP-A by smoking/COPD is in agreement with previous investigations though there are many controversial findings as well. Plasma/serum SP-A levels have been reported to be elevated in COPD in a Japanese cohort of smokers and patients with COPD and pulmonary thromboembolism [6]. These findings are in line with other investigations of the levels of SP-A in the circulating blood [33-36]. In a recent proteomic study of pulmonary tissue, SP-A was found to be one of the most markedly elevated spots when investigated by two dimensional electrophoresis, i.e. its presence in the spots was confirmed by mass spectrometry [8]. There are some opposite findings with respect to the circulating SP-A levels in smokers and/or COPD determined in serum [20,37], lung tissue [38] or bronchoalveolar lavage (BAL) [15,16,39]. In some of those studies, SP-A had been either investigated by a non-quantitative immunohistochemical technique or from BAL, which is problematic due to its invasive nature and collapse of the airways in COPD. Very few studies have investigated the effect of age on the levels of potential plasma biomarkers, this report has concentrated on these changes, especially with respect to SP-A, since these may be remarkable and need to be taken into careful consideration in all corresponding investigations.

Genotype and age of the patients have been shown to have an influence on the SP-A levels [40,41] and some SP-A alleles may increase the risk for development of COPD [42]. In the study of Ohlmeier [8], SP-A was confirmed to represent SP-A2, but the assay used in the present study is known to detect both SP-A1 and SP-A2 isoforms. Further studies will be necessary to investigate the long term changes in plasma SP-A levels, and also if SP-A1 and SP-A2 predict disease progression or whether these changes are related to smoking alone.

SP-D has been proposed to represent a marker for COPD, especially predicting its exacerbations [17]. However, there are studies indicating that serum SP-D is not changed [6] in smokers/COPD or that its levels in BAL are reduced in smokers [15,16,43] and patients with COPD [44]. In the present study, plasma SP-D levels did not differ between the young and old age groups; neither were they different between OS and COPD. These data are in agreement with our earlier results showing no significant changes in lung tissue SP-D in COPD [8]. Overall, SP-D revealed only slight variability, possibly some elevation due to smoking, but the changes were smaller than those observed with SP-A.

MMP-9 and TIMP-1 have been postulated to be associated with COPD and its development [4,5,26,27]. This was the reason why MMP-9 and TIMP-1 were included in the present study. This study confirms previous investigations that the MMP-9 level is increased in long-term smokers [5,45], but as far as we are aware, there are no studies which have investigated the levels of MMP-9 or TIMP-1 in different age groups of non-smokers or smokers. This study detected no significant difference in plasma MMP-9 between YNS and YS but as shown earlier, the changes observed in MMP-9 and TIMP-1 were mostly attributable to smoking. These data also suggest that short-term smoking (<10 years) in young healthy subjects does not significantly modify the levels of circulating MMP-9 or TIMP-1. However, long-term smoking (>10 years) increased both plasma levels of MMP-9 and MMP-9/TIMP-1, perhaps reflecting increased systemic inflammation, though active remodelling in the airways is evidently more prominent. Since there are multiple MMPs and TIMPs, the MMP-9/TIMP-1 ratio alone does not reflect the overall protease/antiprotease balance in the lung. Many studies have been conducted on MMPs and the protease/antiprotease balance in COPD, but it is still unclear if these changes predict COPD in the longitudinal setting.

The weakness of this study is that in the young age group, most of the individuals were men. Therefore we compared the results between the genders in the middle aged/elderly group but could detect no significant differences between the genders with respect to the levels of SP-D, MMP-9 or TIMP-1. Only the plasma SP-A level was significantly higher in middle-aged/older non-smoking women when compared to the men (p = 0.012). This was also a cross-sectional study, and therefore the value of these proteins in COPD will require further prospective investigations. This study has significant strengths i.e. none of the non-smokers or smokers had any other exposures. The chronic smokers suffering from COPD, were taking no medications for COPD since they had newly diagnosed COPD and none of them had any other diseases or were taking any medications. This does not exclude the existence of cardiovascular or other organ involvements, though all subjects considered themselves as healthy [28].

## Conclusions

In conclusion, the plasma level of SP-A may be a promising marker for COPD, it displayed age related changes and exhibited a significant elevation due to smoking, but prospective studies will be needed to elucidate the significance of SP-A and also potential new markers of COPD. Two prospective studies are ongoing in our laboratory [28,46], where potential markers are being evaluated and will be tested within the next two and five years. These and other potential markers may also prove to be important in young smokers, especially when evaluating early lung damage in cases when smoking or other environmental exposures are combined with impaired lung development and/or other abnormalities of the lung.

## List of abbreviations

B: regression coefficient; BMI: body mass index; CI: confidence interval; COPD: chronic obstructive pulmonary disease; EIA: enzyme immunoassay; ELISA: enzyme-linked immunosorbent assay; FEV1: forced expiratory volume in one second; FVC: forced vital capacity; GOLD: the Global initiative for chronic Obstructive Lung Disease; MMP: matrix metalloproteinase; ONS: middle aged/elderly (old) non-smokers; OS: middle aged/elderly (old) smokers; ROC: receiver operating characteristic; TIMP: tissue inhibitor of metalloproteinase; SD: standard deviation; SE: standard error; SP: surfactant protein; YNS: young non-smokers; YS: young smokers.

## Competing interests

The authors declare that they have no competing interests.

## Authors' contributions

HI participated in the planning of the study, performed part of the laboratory analysis, together with PN made statistical analyses and interpretation of data, prepared the tables, conducted the literature research and participated in the writing process. WM prepared the figures and participated in the writing process. TT participated in the recruitment and interview of the subjects and their characterization and was responsible for the lung function analysis. NL participated in the laboratory analysis and in the writing process. PN contributed to the statistical analyses and interpretation of data. HK was responsible for SP-A analysis and revision of the manuscript. NI calculated the ROC curves and prepared the figures. VK is the principal investigator, has designed the study, conducted the literature research and had a major role in writing process. All authors read and approved the final manuscript.

## Pre-publication history

The pre-publication history for this paper can be accessed here:

http://www.biomedcentral.com/1471-2466/11/19/prepub

## References

[B1] Fletcher C Peto R The natural history of chronic airflow obstruction Br Med J 1977 1 1645 1648 10.1136/bmj.1.6077.1645 871704 PMC1607732

[B2] Rabe KF Hurd S Anzueto A Barnes PJ Buist SA Calverley P Fukuchi Y Jenkins C Rodriguez-Roisin R van Weel C Zielinski J Global strategy for the diagnosis, management, and prevention of chronic obstructive pulmonary disease: GOLD executive summary Am J Respir Crit Care Med 2007 176 532 555 10.1164/rccm.200703-456SO 17507545

[B3] Scanlon PD Connett JE Waller LA Altose MD Bailey WC Buist AS Smoking cessation and lung function in mild-to-moderate chronic obstructive pulmonary disease. The Lung Health Study Am J Respir Crit Care Med 2000 161 381 390 10673175 10.1164/ajrccm.161.2.9901044

[B4] Beeh KM Beier J Kornmann O Buhl R Sputum matrix metalloproteinase-9, tissue inhibitor of metalloprotinease-1, and their molar ratio in patients with chronic obstructive pulmonary disease, idiopathic pulmonary fibrosis and healthy subjects Respir Med 2003 97 634 639 10.1053/rmed.2003.1493 12814147

[B5] Ilumets H Rytilä P Demedts I Brusselle GG Sovijärvi A Myllärniemi M Sorsa T Kinnula VL Matrix metalloproteinases -8, -9 and -12 in smokers and patients with stage 0 COPD Int J Chron Obstruct Pulmon Dis 2007 2 369 379 18229576 PMC2695187

[B6] Kobayashi H Kanoh S Motoyoshi K Serum surfactant protein-A, but not surfactant protein-D or KL-6, can predict preclinical lung damage induced by smoking Biomarkers 2008 13 385 392 10.1080/13547500801903651 18595202

[B7] Louhelainen N Stark H Mazur W Rytilä P Djukanovic R Kinnula VL Elevation of sputum matrix metalloproteinase-9 persists up to 6 months after smoking cessation: a research study BMC Pulm Med 2010 10 13 10.1186/1471-2466-10-13 20226090 PMC2841651

[B8] Ohlmeier S Vuolanto M Toljamo T Vuopala K Salmenkivi K Myllärniemi M Kinnula VL Proteomics of human lung tissue identifies surfactant protein A as a marker of chronic obstructive pulmonary disease J Proteome Res 2008 7 5125 5132 10.1021/pr800423x 19367700

[B9] Sin DD Leung R Gan WQ Man SP Circulating surfactant protein D as a potential lung-specific biomarker of health outcomes in COPD: a pilot study BMC Pulm Med 2007 7 13 10.1186/1471-2466-7-13 17922919 PMC2096624

[B10] Kishore U Greenhough TJ Waters P Shrive AK Ghai R Kamran MF Bernal AL Reid KB Madan T Chakraborty T Surfactant proteins SP-A and SP-D: structure, function and receptors Mol Immunol 2006 43 1293 1315 10.1016/j.molimm.2005.08.004 16213021

[B11] Mason RJ Greene K Voelker DR Surfactant protein A and surfactant protein D in health and disease Am J Physiol 1998 275 L1 13 9688929 10.1152/ajplung.1998.275.1.L1

[B12] Whitsett JA Surfactant proteins in innate host defense of the lung Biol Neonate 2005 88 175 180 10.1159/000087580 16210839

[B13] Otto-Verberne CJ Ten Have-Opbroek AA Franken C Hermans J Dijkman JH Protective effect of pulmonary surfactant on elastase-induced emphysema in mice Eur Respir J 1992 5 1223 1230 1486969

[B14] Wirtz HR Schmidt M Acute influence of cigarette smoke on secretion of pulmonary surfactant in rat alveolar type II cells in culture Eur Respir J 1996 9 24 32 10.1183/09031936.96.09010024 8834329

[B15] Honda Y Takahashi H Kuroki Y Akino T Abe S Decreased contents of surfactant proteins A and D in BAL fluids of healthy smokers Chest 1996 109 1006 1009 10.1378/chest.109.4.1006 8635323

[B16] Betsuyaku T Kuroki Y Nagai K Nasuhara Y Nishimura M Effects of ageing and smoking on SP-A and SP-D levels in bronchoalveolar lavage fluid Eur Respir J 2004 24 964 970 10.1183/09031936.04.00064004 15572540

[B17] Lomas DA Silverman EK Edwards LD Locantore NW Miller BE Horstman DH Tal-Singer R Serum surfactant protein D is steroid sensitive and associated with exacerbations of COPD Eur Respir J 2009 34 95 102 10.1183/09031936.00156508 19164344

[B18] Antoniu SA Inhaled corticosteroids in COPD: systemic effects of a local therapy? Expert Opin Pharmacother 2008 9 3271 3273 10.1517/14656560802591406 19040347

[B19] Sin DD Man SF Marciniuk DD Ford G FitzGerald M Wong E York E Mainra RR Ramesh W Melenka LS Wilde E Cowie RL Williams D Gan WQ Rousseau R The effects of fluticasone with or without salmeterol on systemic biomarkers of inflammation in chronic obstructive pulmonary disease Am J Respir Crit Care Med 2008 177 1207 1214 10.1164/rccm.200709-1356OC 18310480

[B20] Mutti A Corradi M Goldoni M Vettori MV Bernard A Apostoli P Exhaled metallic elements and serum pneumoproteins in asymptomatic smokers and patients with COPD or asthma Chest 2006 129 1288 1297 10.1378/chest.129.5.1288 16685021 PMC1472634

[B21] LeVine AM Whitsett JA Gwozdz JA Richardson TR Fisher JH Burhans MS Korfhagen TR Distinct effects of surfactant protein A or D deficiency during bacterial infection on the lung J Immunol 2000 165 3934 3940 11034401 10.4049/jimmunol.165.7.3934

[B22] Ramadas RA Wu L LeVine AM Surfactant protein A enhances production of secretory leukoprotease inhibitor and protects it from cleavage by matrix metalloproteinases J Immunol 2009 182 1560 1567 19155504 10.4049/jimmunol.182.3.1560

[B23] Vazquez de Lara LG Umstead TM Davis SE Phelps DS Surfactant protein A increases matrix metalloproteinase-9 production by THP-1 cells Am J Physiol Lung Cell Mol Physiol 2003 285 L899 L906 12842807 10.1152/ajplung.00082.2003

[B24] Wert SE Yoshida M LeVine AM Ikegami M Jones T Ross GF Fisher JH Korfhagen TR Whitsett JA Increased metalloproteinase activity, oxidant production, and emphysema in surfactant protein D gene-inactivated mice Proc Natl Acad Sci USA 2000 97 5972 5977 10.1073/pnas.100448997 10801980 PMC18543

[B25] Gadek JE Pacht ER The protease-antiprotease balance within the human lung: implications for the pathogenesis of emphysema Lung 1990 168 Suppl 552 564 10.1007/BF02718178 2117164

[B26] Barnes PJ Genetics and pulmonary medicine. 9. Molecular genetics of chronic obstructive pulmonary disease Thorax 1999 54 245 252 10.1136/thx.54.3.245 10325902 PMC1745439

[B27] Nagase H Visse R Murphy G Structure and function of matrix metalloproteinases and TIMPs Cardiovasc Res 2006 69 562 573 10.1016/j.cardiores.2005.12.002 16405877

[B28] Toljamo T Kaukonen M Nieminen P Kinnula VL Early detection of COPD combined with individualized counselling for smoking cessation: a two-year prospective study Scand J Prim Health Care 2010 28 41 46 10.3109/02813431003630105 20331388 PMC3440614

[B29] Hamari A Toljamo T Nieminen P Kinnula VL High frequency of chronic cough and sputum production with lowered exercise capacity in young smokers Ann Med 2010 42 512 520 10.3109/07853890.2010.505933 20662762

[B30] GOLD Global strategy for diagnosis, management and prevention 2009 http://www.goldcopd.com

[B31] Viljanen AA Halttunen PK Kreus KE Viljanen BC Spirometric studies in non-smoking, healthy adults Scand J Clin Lab Invest Suppl 1982 159 5 20 6957974

[B32] Takahashi H Fujishima T Koba H Murakami S Kurokawa K Shibuya Y Shiratori M Kuroki Y Abe S Serum surfactant proteins A and D as prognostic factors in idiopathic pulmonary fibrosis and their relationship to disease extent Am J Respir Crit Care Med 2000 162 1109 1114 10988138 10.1164/ajrccm.162.3.9910080

[B33] Robin M Dong P Hermans C Bernard A Bersten AD Doyle IR Serum levels of CC16, SP-A and SP-B reflect tobacco-smoke exposure in asymptomatic subjects Eur Respir J 2002 20 1152 1161 10.1183/09031936.02.02042001 12449168

[B34] Nomori H Horio H Fuyuno G Kobayashi R Morinaga S Suemasu K Serum surfactant protein A levels in healthy individuals are increased in smokers Lung 1998 176 355 361 10.1007/PL00007617 9780293

[B35] Kida K Oda H Yamano Y Kagawa J Effects of cigarette smoking on the serum concentration of lung surfactant protein A (SP-A) Eur Respir J 1997 10 2124 2126 10.1183/09031936.97.10092124 9311515

[B36] Behera D Balamugesh T Venkateswarlu D Gupta A Majumdar S Serum surfactant protein-A levels in chronic bronchitis and its relation to smoking Indian J Chest Dis Allied Sci 2005 47 13 17 15704710

[B37] Greene KE King TE Kuroki Y Bucher-Bartelson B Hunninghake GW Newman LS Nagae H Mason RJ Serum surfactant proteins-A and -D as biomarkers in idiopathic pulmonary fibrosis Eur Respir J 2002 19 439 446 10.1183/09031936.02.00081102 11936520

[B38] Vlachaki EM Koutsopoulos AV Tzanakis N Neofytou E Siganaki M Drositis I Moniakis A Schiza S Siafakas NM Tzortzaki EG Altered surfactant protein-A expression in type II pneumocytes in COPD Chest 2010 137 37 45 10.1378/chest.09-1029 19741063

[B39] Fujishima T Takahashi H Abe S Cytokines and surfactant as a factor of onset and progression of COPD Nippon Rinsho 1999 57 1976 1981 10497393

[B40] Floros J Human surfactant protein A (SP-A) variants: why so many, why such a complexity? Swiss Med Wkly 2001 131 87 90 11416882 10.4414/smw.2001.06136

[B41] Tagaram HR Wang G Umstead TM Mikerov AN Thomas NJ Graff GR Hess JC Thomassen MJ Kavuru MS Phelps DS Floros J Characterization of a human surfactant protein A1 (SP-A1) gene-specific antibody; SP-A1 content variation among individuals of varying age and pulmonary health Am J Physiol Lung Cell Mol Physiol 2007 292 L1052 L1063 10.1152/ajplung.00249.2006 17189324

[B42] Guo X Lin HM Lin Z Montano M Sansores R Wang G DiAngelo S Pardo A Selman M Floros J Surfactant protein gene A, B, and D marker alleles in chronic obstructive pulmonary disease of a Mexican population Eur Respir J 2001 18 482 490 10.1183/09031936.01.00043401 11589345

[B43] More JM Voelker DR Silveira LJ Edwards MG Chan ED Bowler RP Smoking reduces surfactant protein D and phospholipids in patients with and without chronic obstructive pulmonary disease BMC Pulm Med 2010 10 53 10.1186/1471-2466-10-53 20973980 PMC2987951

[B44] Sims MW Tal-Singer RM Kierstein S Musani AI Beers MF Panettieri RA Haczku A Chronic obstructive pulmonary disease and inhaled steroids alter surfactant protein D (SP-D) levels: a cross-sectional study Respir Res 2008 9 13 10.1186/1465-9921-9-13 18226251 PMC2249580

[B45] Lim S Roche N Oliver BG Mattos W Barnes PJ Chung KF Balance of matrix metalloprotease-9 and tissue inhibitor of metalloprotease-1 from alveolar macrophages in cigarette smokers. Regulation by interleukin-10 Am J Respir Crit Care Med 2000 162 1355 1360 11029344 10.1164/ajrccm.162.4.9910097

[B46] Laitinen T Hodgson U Kupiainen H Tammilehto L Haahtela T Kilpeläinen M Lindqvist A Kinnula VL Real-world clinical data identifies gender-related profiles in chronic obstructive pulmonary disease COPD 2009 6 256 262 10.1080/15412550903051799 19811384

